# Supplementary material for: Tuning the Stability of DNA Tetrahedra with Base Stacking Interactions
Source: Nano Lett. 2025 Feb 20;25(9):3605–12. doi: 10.1021/acs.nanolett.4c06548 (PMC11887421; doi:10.1021/acs.nanolett.4c06548)
Supplement: Supplementary file 1 — nl4c06548_si_001.pdf [file nl4c06548_si_001.pdf]

Supplementary Information for:

Tuning the stability of DNA tetrahedra with base-stacking interactions

Jibin Abraham Punnoose,[1^*] Dadrian Cole,[1,2^] Tristan Melfi,[3] Vinod Morya,[1] Bharath Raj Madhanagopal,[1] Alan A. Chen,[1,3] , Sweta Vangaveti,[1] Arun Richard Chandrasekaran,[1,4] and Ken Halvorsen[1*]

[1]The RNA Institute, University at Albany, State University of New York, Albany, NY, 12222 USA.
[2]Department of Biological Sciences, University at Albany, State University of New York, Albany, NY, 12222 USA.
[3]Department of Chemistry, University at Albany, State University of New York, Albany, NY, 12222 USA.
[4]Department of Nanoscale Science and Engineering, University at Albany, State University of New York, Albany, NY, 12222 USA.

^These authors contributed equally to this work

Correspondence: khalvorsen@albany.edu; jabrahampunnoose@albany.edu

Table of Contents:

Supplementary Methods: Tetrahedron assembly, melting and analysis, MD
Table S1: List of oligonucleotides
Table S2: Combination of oligonucleotides to form the various tetrahedra
Figure S1: Gel images of tetrahedrons with 5nt and 6nt sticky end
Figure S2: Annealing protocol for DNA tetrahedra.
Figure S3: Gel images of tetrahedron with A|A base-stack at various temperatures
Figure S4: Gel images of tetrahedron with A|C base-stack at various temperatures
Figure S5: Gel images of tetrahedron with A|G base-stack at various temperatures
Figure S6: Gel images of tetrahedron with A|T base-stack at various temperatures
Figure S7: Gel images of tetrahedron with C|A base-stack at various temperatures
Figure S8: Gel images of tetrahedron with C|C base-stack at various temperatures
Figure S9: Gel images of tetrahedron with C|G base-stack at various temperatures
Figure S10: Gel images of tetrahedron with C|T base-stack at various temperatures
Figure S11: Gel images of tetrahedron with G|A base-stack at various temperatures
Figure S12: Gel images of tetrahedron with G|C base-stack at various temperatures
Figure S13: Gel images of tetrahedron with G|G base-stack at various temperatures
Figure S14: Gel images of tetrahedron with G|T base-stack at various temperatures
Figure S15: Gel images of tetrahedron with T|A base-stack at various temperatures
Figure S16: Gel images of tetrahedron with T|C base-stack at various temperatures
Figure S17: Gel images of tetrahedron with T|G base-stack at various temperatures
Figure S18: Gel images of tetrahedron with T|T base-stack at various temperatures
Figure S19: Gel images of tetrahedron with G|T and T|T base-stack at various temperatures
Figure S20: Gel images of tetrahedron with 2 A|A base-stack & 4-nt sticky end at various temperatures
Figure S21: Triplicate melting experiments with tetrahedron with 1 T|T stack and a 5 nucleotide sticky end
Figure S22: Gel images showing formation of different 6 and 4-nt tetrahedron with various base-stacks
Figure S23: Thermal stability difference between tetrahedron with T|T stacks and A|A stacks.
Figure S24: Dynamic light scattering of tetrahedra with T|G and A|A stacks.

Supplementary Methods

Tetrahedra construction

To assemble the DNA tetrahedron, desalted L, M and S strands (Table S1) were mixed in a 1:5:5 ratio at a final concentration of 30nM in Tris-acetate-EDTA-Mg^{2+} buffer (40mM Tris base, pH 8.0; 20mM acetic acid; and 12.5mM magnesium acetate). The DNA samples were then annealed by placing them in a 2L water bath within a Styrofoam box, which allowed for their gradual cooling from 90°C to room temperature over a 48-hour period. To assemble DNA tetrahedra with different base stacks varying strand combinations were used (Table S2).

For one set of experiments, a thermal cycler was used to assemble the tetrahedra. To program the thermal cycler, we first measured the cooling rate of the thermal bath in Styrofoam using a thermometer (Figure S2). The thermal cycler was then programmed with the following cooling profile: 85 °C to 75 °C at a rate of -1 °C per 9 minutes, 75 °C to 60 °C at a rate of -1 °C per 12 minutes, 60 °C to 51 °C at a rate of -1 °C per 20 minutes, 51 °C to 43 °C at a rate of -1 °C per 20 minutes, 43 °C to 38 °C at a rate of -1 °C per 36 minutes, 38 °C to 34 °C at a rate of -1 °C per 45 minutes, 34 °C to 32 °C at a rate of -1 °C per 90 minutes, 32 °C to 31 °C at a rate of -1 °C per 180 minutes, 31 °C to 30 °C at a rate of -1 °C per 180 minutes, and 30 °C to 23 °C at a rate of -1 °C per 200 minutes.

Thermal analysis

To analyze the thermal stability of DNA tetrahedra, 5 µL of each tetrahedron was placed in nine tubes and incubated in a T100 Thermal Cycler (Bio-Rad) for one hour at predetermined experimental temperatures. After incubation, 5 µL of the samples were mixed with 1 µL of a glycerol based gel loading dye, and 5 µL total was used for each sample. Samples were run at 4 °C on 4% non-denaturing polyacrylamide (29:1 acrylamide/bisacrylamide) gels at 100V for one hour. Following electrophoresis, gels were stained in 50 mL of 1x Tris-acetate-EDTA-Mg^{2+} buffer and 5 µL of 10,000X GelRed (Biotium) before imaging. Imaging was done with an Azure Biosystems Imager using the default settings for Ethidium Bromide ultraviolet illumination.

The intact fraction of tetrahedra in the polyacrylamide gel was measured using the Bio-Rad ImageLab Software. The bands corresponding to the DNA tetrahedron with varied base stacks at different temperatures were quantified and normalized against the band at room temperature. These normalized values were plotted in OriginLab and the data was fitted using a Boltzmann function, $f(x) = (A2 - A1)\ (1 + e(x - x_0)/dx) + A1$, where A1 is the lower asymptote, A2 is the upper asymptote, x is the independent variable, x_0 is the inflection point, and d*x is* a parameter associated with the width of the sigmoidal curve. The thermal stability was determined by using individual x_0 values which is typically considered to represent the midpoint of the transition from one state to another, commonly known as the melting point. Fitting uncertainty from each replicate was used to determine the error represented as the uncertainty of the average melting temperatures from three individual measurements.

MD simulations

To investigate the difference in stability between the 2 stacking configurations we simulated 2 nicked duplexes: one with A|T stacking and the other with T|A stacking at the nicked interface in all atom molecular dynamics simulations. After modelling the two duplexes in Molecular Operating Environment (MOE), they were then simulated using GROMACS 2019.4 (1) for 250 ns at temperatures between 300 K to 400 K in 20 K increments, performing a total of simulations. Each system was neutralized by adding K^+ and Cl^- ions. The tumuc1 force-field (2) was used with the accompanying ion parameters for this system.

All of the molecular dynamics simulations incorporated the leap-frog algorithm to integrate the equations of motion. The systems used the velocity rescaling thermostat to maintain the temperature (3). Pressure was maintained at 1 atm using Parrinello-Rahman barostat (4) for the equilibration. Long-range electrostatic interactions were calculated using particle mesh Ewald (PME) algorithm with a cut-off of 1.0 nm (5) Lennard-Jones interactions were also truncated at 1.0 nm. Water molecules were represented using the TIP3P model (6), and the LINCS algorithm was used to constrain the motion of hydrogen atoms bonded to heavy atoms (7). Each system was subjected to energy minimization to prevent any overlap of atoms, followed by 100 ps temperature and pressure equilibration, leading to the final production run of 250 ns. The simulations were visualized using PyMOL and the MolecularNodes Blender plugin. The rmsd and distance tools from GROMACS (1) were used for analysis. The distance between the bases was defined as the distance between the center of mass of the heavy atoms on the nucleobases. Nucleotides were considered stacked if this distance was less than 0.3 nm.

References:

1. Abraham,M.J., Murtola,T., Schulz,R., Páll,S., Smith,J.C., Hess,B. and Lindahl,E. (2015) GROMACS: High performance molecular simulations through multi-level parallelism from laptops to supercomputers. *SoftwareX*, 1-2, 19–25.

2. Liebl, K., & Zacharias, M. (2021). Tumuc1: A New Accurate DNA Force Field Consistent with High-Level Quantum Chemistry. *Journal of Chemical Theory and Computation*, *17*(11), 7096–7105. https://doi.org/10.1021/acs.jctc.1c00682

3. Bussi,G., Donadio,D. and Parrinello,M. (2007) Canonical sampling through velocity rescaling. *J. Chem. Phys.*, 126.

4. Parrinello, M., & Rahman, A. (1980). Crystal Structure and pair Potentials: A Molecular-Dynamics study. *Physical Review Letters*, *45*(14), 1196–1199. https://doi.org/10.1103/physrevlett.45.1196

5. Darden,T., York,D. and Pedersen,L. (1993) Particle mesh Ewald: An $N \cdot \log(N)$ method for Ewald sums in large systems. *J. Chem. Phys.*, 98, 10089–10092.

6. Mark,P. and Nilsson,L. (2001) Structure and dynamics of the TIP3P, SPC, and SPC/E water models at 298 K. *J. Phys. Chem. A*, 105, 9954–9960.

7. Hess,B., Bekker,H., Berendsen,H.J.C. and Fraaije,J.G.E.M. (1997) LINCS: A linear constraint solver for molecular simulations. *J. Comput. Chem.*, 18, 1463–1472.

Table S1: List of oligonucleotides

Name	Sequence (5'-3')	Length
Tet L	AGG CAC CAT CGT AGG TTT TTC TTG CCA GGC ACC ATC GTA GGT TTT TCT TGC CAG GCA CCA TCG TAG GTT TTT CTT GCC	78
TET-M-6SE TG	GGC AAC CTG CCT GGC AAG CCT ACG ATG GAC ACG GAT CTG CAT	42
TET-S-6SE TG	TCC GTG TGG TGG CCA TGC AG	20
TET-M-6SE TC	CGC AAC CTG CCT GGC AAG CCT ACG ATG GAC ACG GAT CTG CAT	42
TET-S-6SE TC	TCC GTG TGG TGG CGA TGC AG	20
TET-M-6SE TT	TGC AAC CTG CCT GGC AAG CCT ACG ATG GAC ACG GAT CTG CAT	42
TET-S-6SE TT	TCC GTG TGG TTG CAA TGC AG	20
TET-M-6SE TA	AGC AAC CTG CCT GGC AAG CCT ACG ATG GAC ACG GAT CTG CAT	42
TET-S-6SE TA	TCC GTG TGG TTG CTA TGC AG	20
TET-M-6SE AG	GCA ACC TGC CTG GCA AGC CTA CGA TGG ACA CGG ACT ACG TC	41
TET-S-6SE AG	GTC CGT GTG GTT GCA GAC GTA	21
TET-M-6SE AC	GCA ACC TGC CTG GCA AGC CTA CGA TGG ACA CGG AGT ACG TC	41
TET-S-6SE AC	CTC CGT GTG GTT GCA GAC GTA	21
TET-M-6SE CG	GGC AAC CTG CCT GGC AAG CCT ACG ATG GAC ACG GAT TAC GTC	42
TET-S-6SE CG	TCC GTG TGG TTG CCG ACG TA	20
TET-M-6SE GG	GCA ACC TGC CTG GCA AGC CTA CGA TGG ACA CGG ACC TGC AT	41
TET-S-6SE GG	GTC CGT GTG GTT GCA ATG CAG	21
TET-M-6SE AT	GCA ACC TGC CTG GCA AGC CTA CGA TGG ACA CGG AAT ACG TC	41
TET-S-6SE AT	TTC CGT GTG GTT GCA GAC GTA	21
TET-M-6SE CC	CGC AAC CTG CCT GGC AAG CCT ACG ATG GAC ACG GAT TAC GTC	42
TET-S-6SE CC	TCC GTG TGG TTG CGG ACG TA	20
TET-M-6SE GC	GCA ACC TGC CTG GCA AGC CTA CGA TGG ACA CGG AGC TGC AT	41
TET-S-6SE GC	CTC CGT GTG GTT GCA ATG CAG	21
TET-M-6SE AA	GCA ACC TGC CTG GCA AGC CTA CGA TGG ACA CGG ATT ACG TC	41
TET-S-6SE AA	ATC CGT GTG GTT GCA GAC GTA	21
TET-M-6SE CA	AGC AAC CTG CCT GGC AAG CCT ACG ATG GAC ACG GAT TAC GTC	42
TET-S-6SE CA	TCC GTG TGG TTG CTG ACG TA	20
TET-M-6SE GT	GCA ACC TGC CTG GCA AGC CTA CGA TGG ACA CGG AAC TGC AT	41
TET-S-6SE GT	TTC CGT GTG GTT GCA ATG CAG	21
TET-M-6SE GA	GCA ACC TGC CTG GCA AGC CTA CGA TGG ACA CGG ATC TGC AT	41
TET-S-6SE GA	ATC CGT GTG GTT GCA ATG CAG	21
TET-M-6SE CT	TGC AAC CTG CCT GGC AAG CCT ACG ATG GAC ACG GAT TAC GTC	42
TET-S-6SE CT	TCC GTG TGG TTG CAG ACG TA	20
TET-M-5SE CT	TAG CAA CCT GCC TGG CAA GCC TAC GAT GGA CAC GGA TTC GTC	42
TET-S-5SE CT	TCC GTG TGG TTG CTA GAC GA	20
TET-M-5SE GA	AGC AAC CTG CCT GGC AAG CCT ACG ATG GAC ACG GAT CTG CT	41
TET-S-5SE GA	ATC CGT GTG GTT GCT AAG CAG	21
TET-M-6SE GT+TT	TGC AAC CTG CCT GGC AAG CCT ACG ATG GAC ACG GAA CTG CAT	42
TET-M-5SE-2TT	TCGGA AGCAACC TGCCTGGCAAG CCTAC GATGG ACACGGTAT	42
TET-S-5SE-1TT	TCCGA ATACCGTGT GGTTGCT	21

TET-M-4SE 2AA	AAG CAA CCT GCC TGG CAA GCC TAC GAT GGA CAC GGT ATT CGA	42
TET-S-4SE 2AA	ATA CCG TGT GGT TGC TTT CGA	21
TET-M-4SE 2TT	TCG AAA GCA ACC TGC CTG GCA AGC CTA CGA TGG ACA CGG TAT	42
TET-S-4SE 2TT	TCG AAT ACC GTG TGG TTG CTT	21
TET-M-0SE-2TT	AAGCAACC TGCCTGGCAAG CCTAC GATGG ACACGGTAT	38
TET-S-0SE-2TT	ATACCGTGT GGTTGCTT	17

Table S2: Combination of oligonucleotides to form the tetrahedron with a specific base-stack

Tetrahedron (base-stack)	Sticky end Length	L Strand	M Strand	S Strand
TG	6 nt	Tet L	TET-M-6SE TG	TET-S-6SE TG
TC	6 nt	Tet L	TET-M-6SE TC	TET-S-6SE TC
TT	6 nt	Tet L	TET-M-6SE TT	TET-S-6SE TT
TA	6 nt	Tet L	TET-M-6SE TA	TET-S-6SE TA
AG	6 nt	Tet L	TET-M-6SE AG	TET-S-6SE AG
AC	6 nt	Tet L	TET-M-6SE AC	TET-S-6SE AC
CG	6 nt	Tet L	TET-M-6SE CG	TET-S-6SE CG
GG	6 nt	Tet L	TET-M-6SE GG	TET-S-6SE GG
AT	6 nt	Tet L	TET-M-6SE AT	TET-S-6SE AT
CC	6 nt	Tet L	TET-M-6SE CC	TET-S-6SE CC
GC	6 nt	Tet L	TET-M-6SE GC	TET-S-6SE GC
AA	6 nt	Tet L	TET-M-6SE AA	TET-S-6SE AA
CA	6 nt	Tet L	TET-M-6SE CA	TET-S-6SE CA
GT	6 nt	Tet L	TET-M-6SE GT	TET-S-6SE GT
GA	6 nt	Tet L	TET-M-6SE GA	TET-S-6SE GA
CT	6 nt	Tet L	TET-M-6SE CT	TET-S-6SE CT
CT	5 nt	Tet L	TET-M-5SE CT	TET-S-5SE CT
AG	5 nt	Tet L	TET-M-5SE CT	TET-S-5SE CT
GT + TT	6 nt	Tet L	TET-M-6SE GT+TT	TET-S-6SE GT
AA + AA	4 nt	Tet L	TET-M-4SE 2AA	TET-S-4SE 2AA
T|T	5 nt	Tet L	TET-M-5SE-2TT	Tet-S-5SE-1TT
T|T + TT	4 nt	Tet L	TET-M-4SE 2TT	TET-S-4SE 2TT
Motif	0	Tet L	TET-M-0SE-2TT	TET-S-0SE-2TT

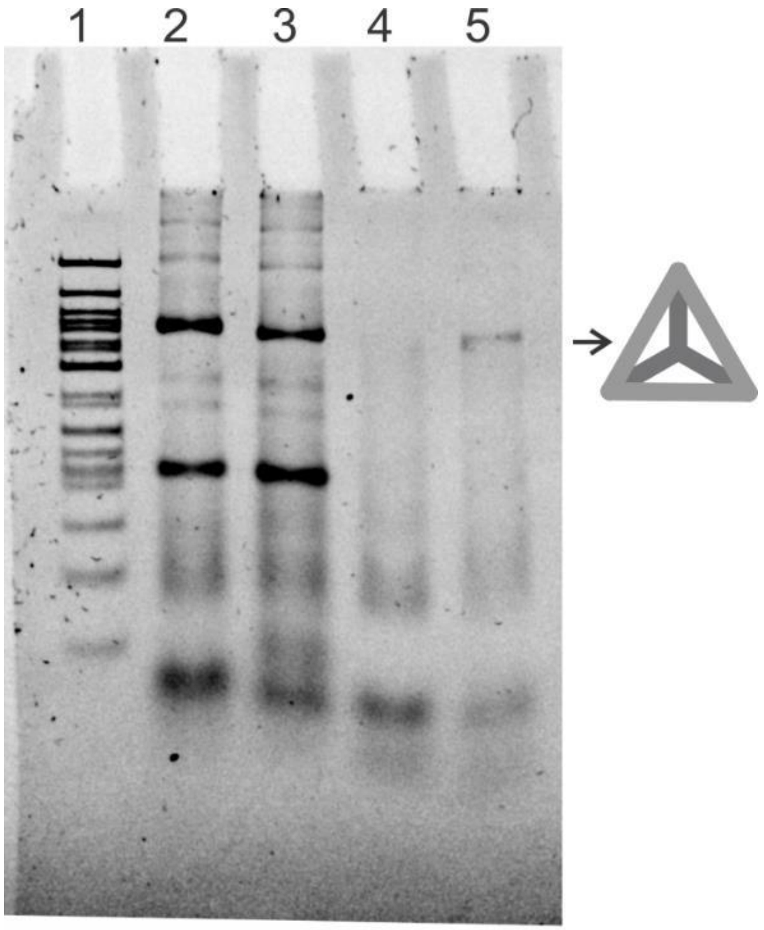

Figure S1: Tetrahedron with 5 and 6 nt sticky ends. Lane 1: 100 bp ladder, Lane 2: Tetrahedron with A|G base-stack and 5 nt sticky end, Lane 3: Tetrahedron with A|G base-stack and 6 nt sticky end, Lane 4: Tetrahedron with C|T base-stack and 5 nt sticky end, and Lane 5: Tetrahedron with C|T base-stack and 6 nt sticky end.

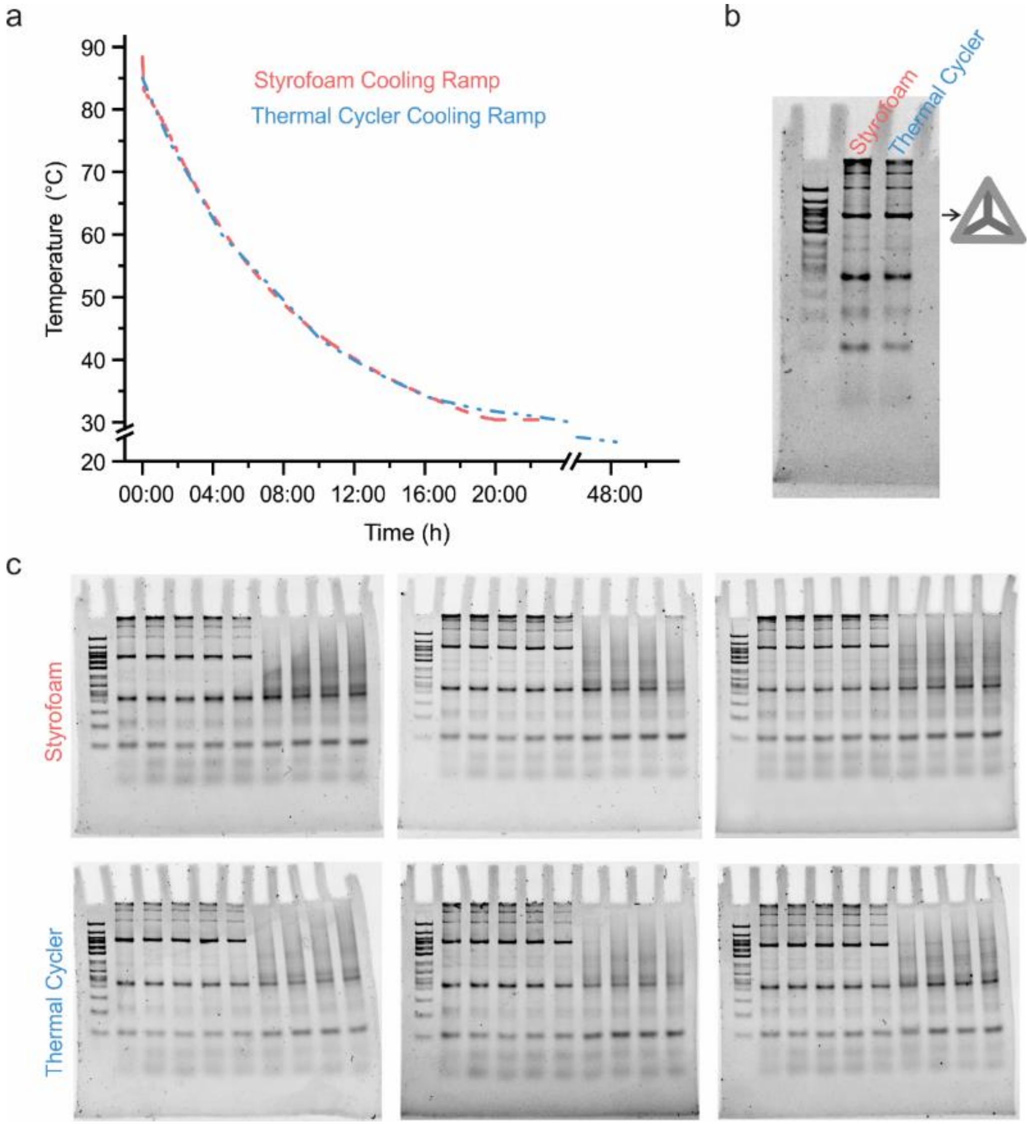

Figure S2: Annealing protocol for DNA tetrahedra. (a) Measured cooling rate of the thermal bath inside the Styrofoam box used for annealing DNA tetrahedra and the thermal cycler cooling profile developed to mimic the process. (b) Comparison of DNA tetrahedron formed using annealing in a Styrofoam and a thermal cycler. (c) Tetrahedron melting comparison using tetrahedron formed using Styrofoam box and a thermal cycler.

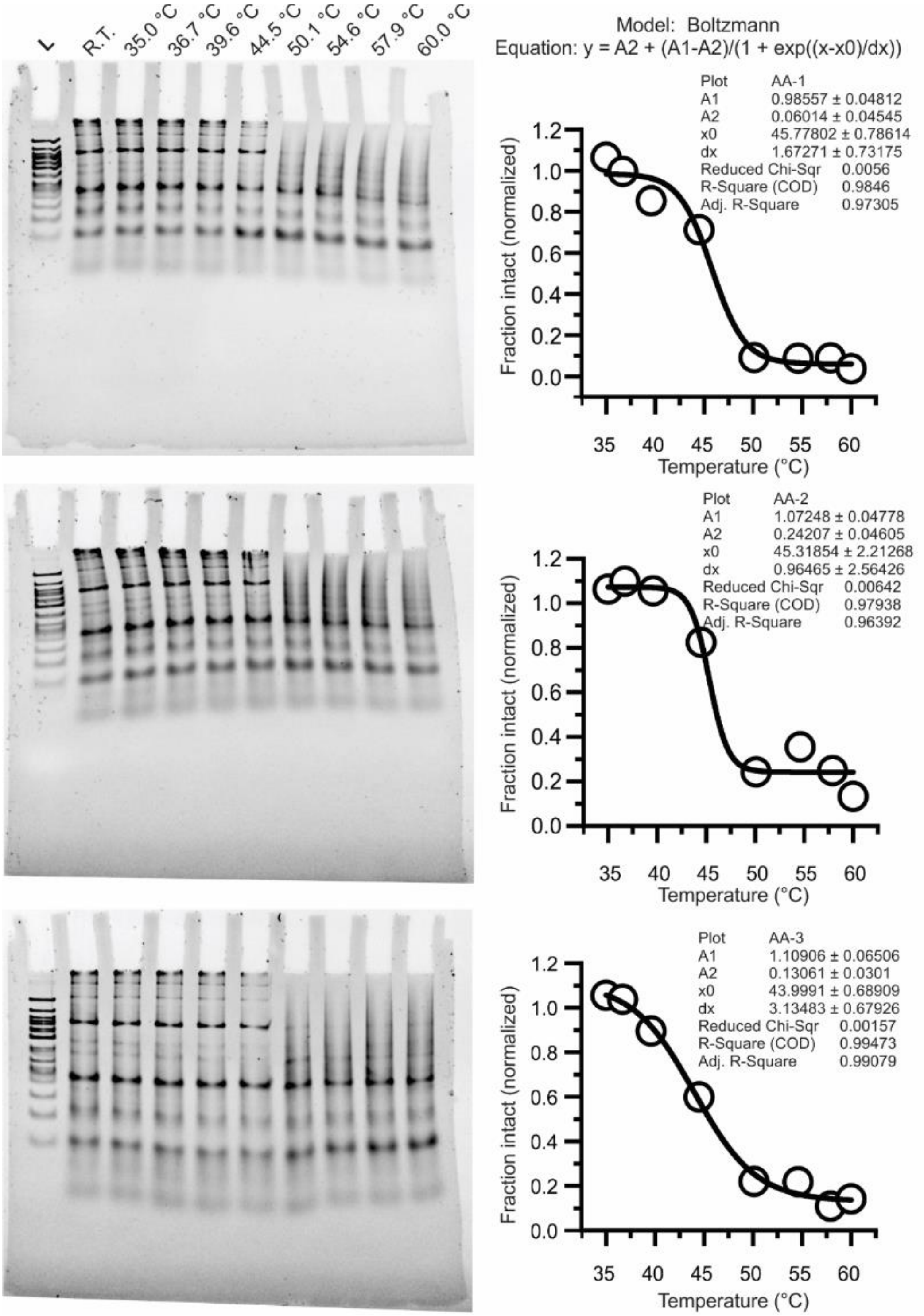

Figure S3: Triplicate melting experiments with tetrahedron with an A|A stack with 6 nucleotide sticky end. The formed nanostructures were incubated at various temperatures for 1 hour as indicated in the lane. The intact fraction of tetrahedron was fitted with Boltzmann model to obtain the melting temperature for each experiment.

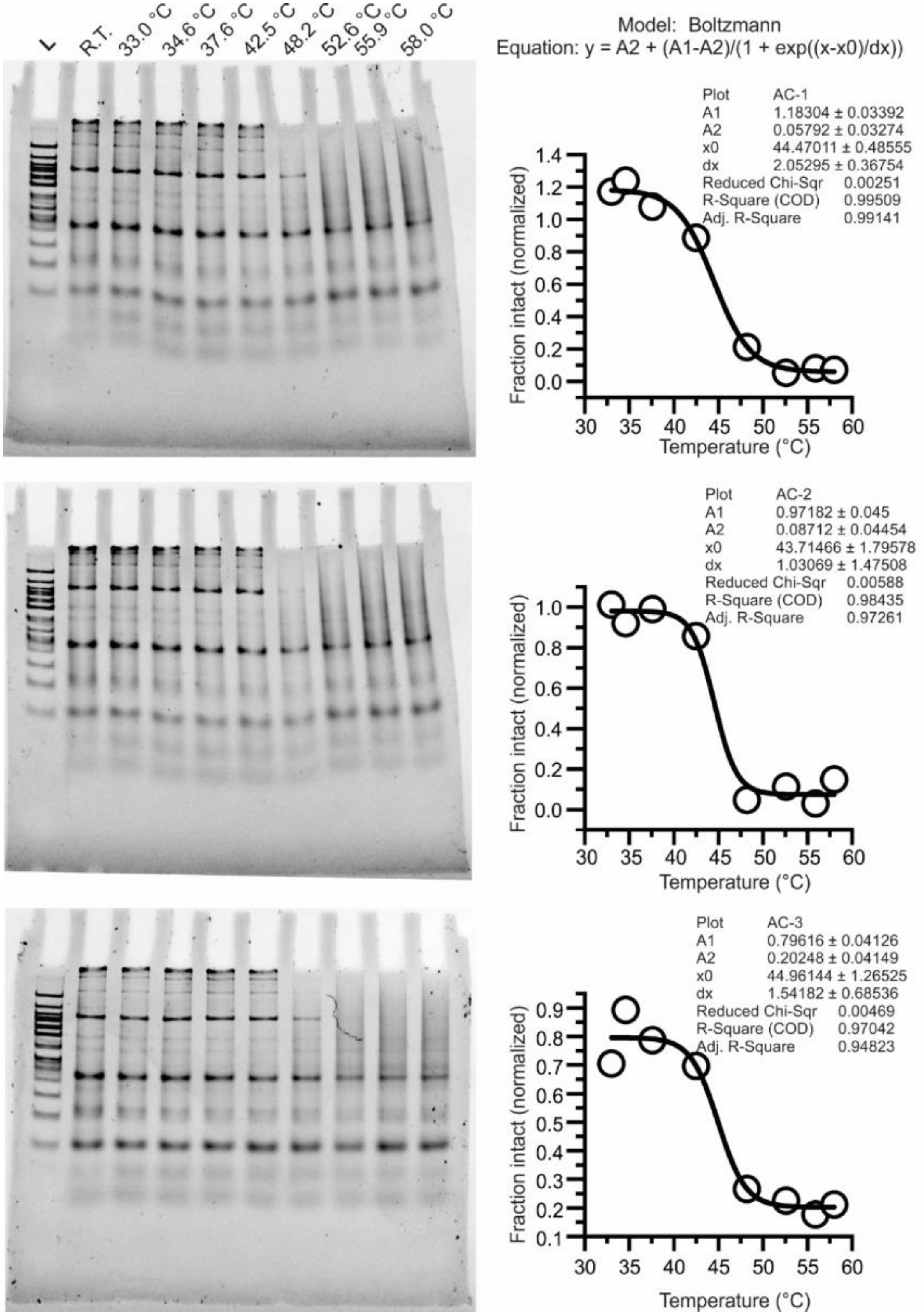

Figure S4: Triplicate melting experiments with tetrahedron with an A|C stack with 6 nucleotide sticky end. The formed nanostructures were incubated at various temperatures for 1 hour as indicated in the lane. The intact fraction of tetrahedron was fitted with Boltzmann model to obtain the melting temperature for each experiment.

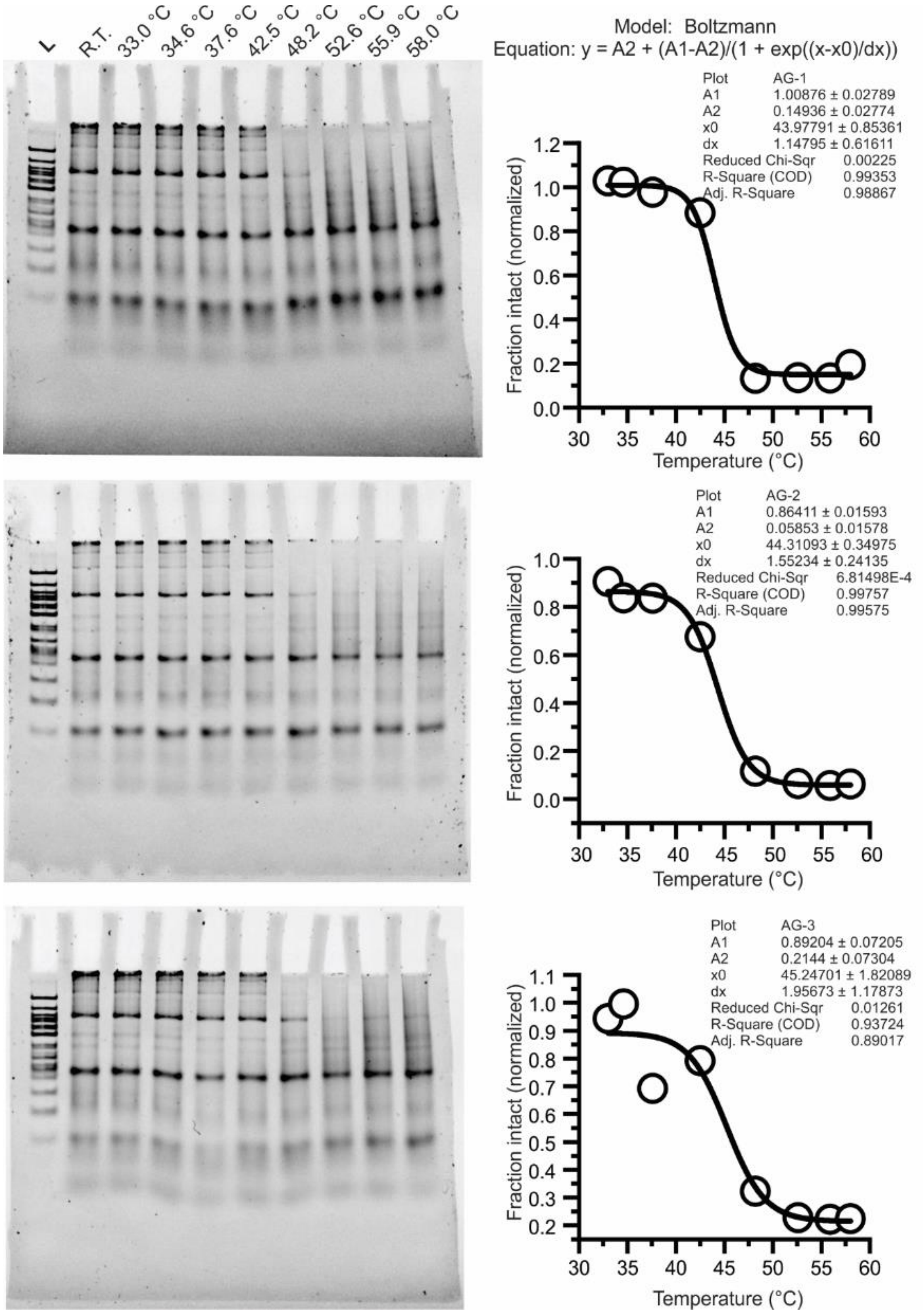

Figure S5: Triplicate melting experiments with tetrahedron with an A|G stack with 6 nucleotide sticky end. The formed nanostructures were incubated at various temperatures for 1 hour as indicated in the lane. The intact fraction of tetrahedron was fitted with Boltzmann model to obtain the melting temperature for each experiment.

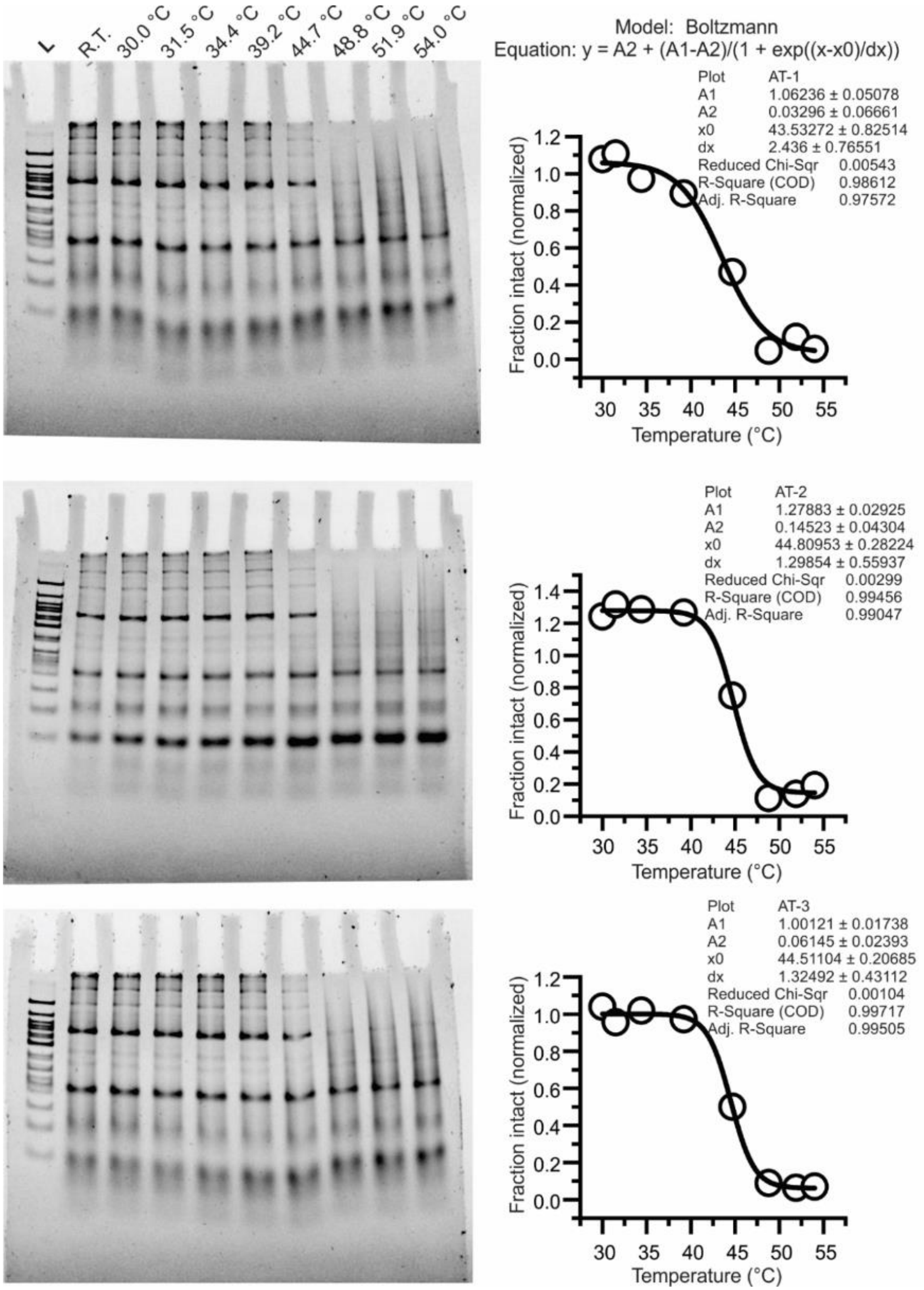

Figure S6: Triplicate melting experiments with tetrahedron with an A|T stack with 6 nucleotide sticky end. The formed nanostructures were incubated at various temperatures for 1 hour as indicated in the lane. The intact fraction of tetrahedron was fitted with Boltzmann model to obtain the melting temperature for each experiment.

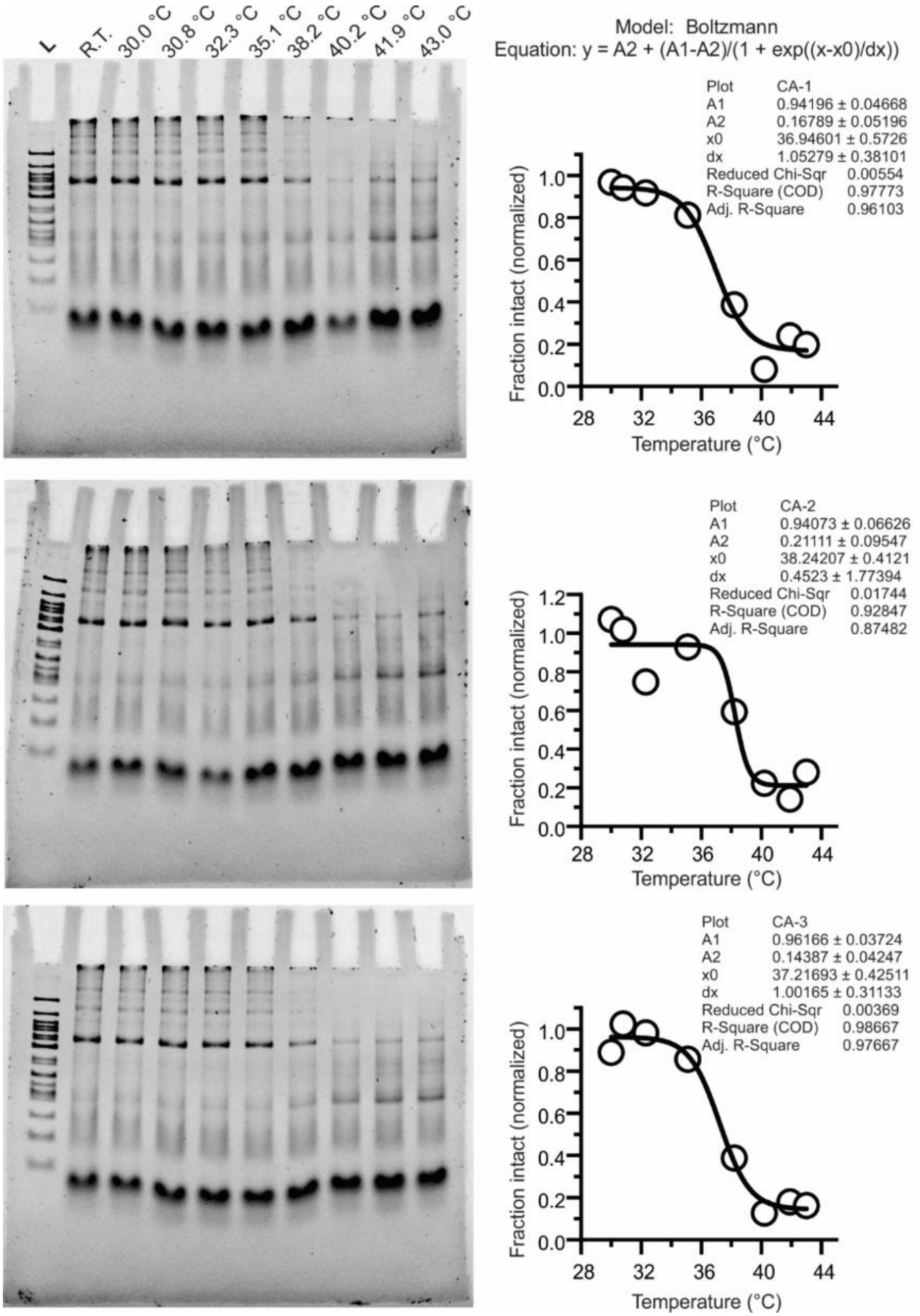

Figure S7: Triplicate melting experiments with tetrahedron with a C|A stack with 6 nucleotide sticky end. The formed nanostructures were incubated at various temperatures for 1 hour as indicated in the lane. The intact fraction of tetrahedron was fitted with Boltzmann model to obtain the melting temperature for each experiment.

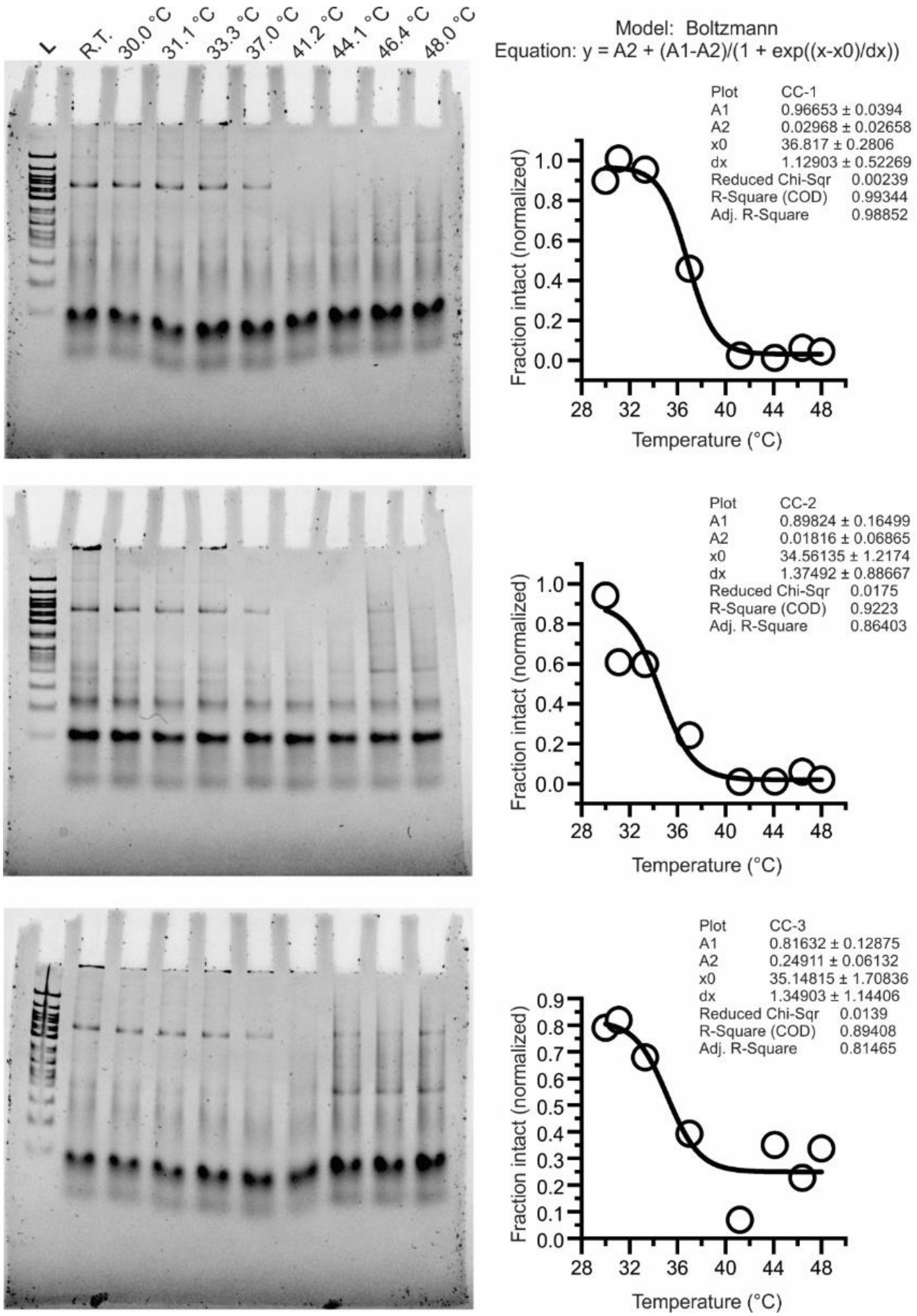

Figure S8: Triplicate melting experiments with tetrahedron with a C|C stack with 6 nucleotide sticky end. The formed nanostructures were incubated at various temperatures for 1 hour as indicated in the lane. The intact fraction of tetrahedron was fitted with Boltzmann model to obtain the melting temperature for each experiment.

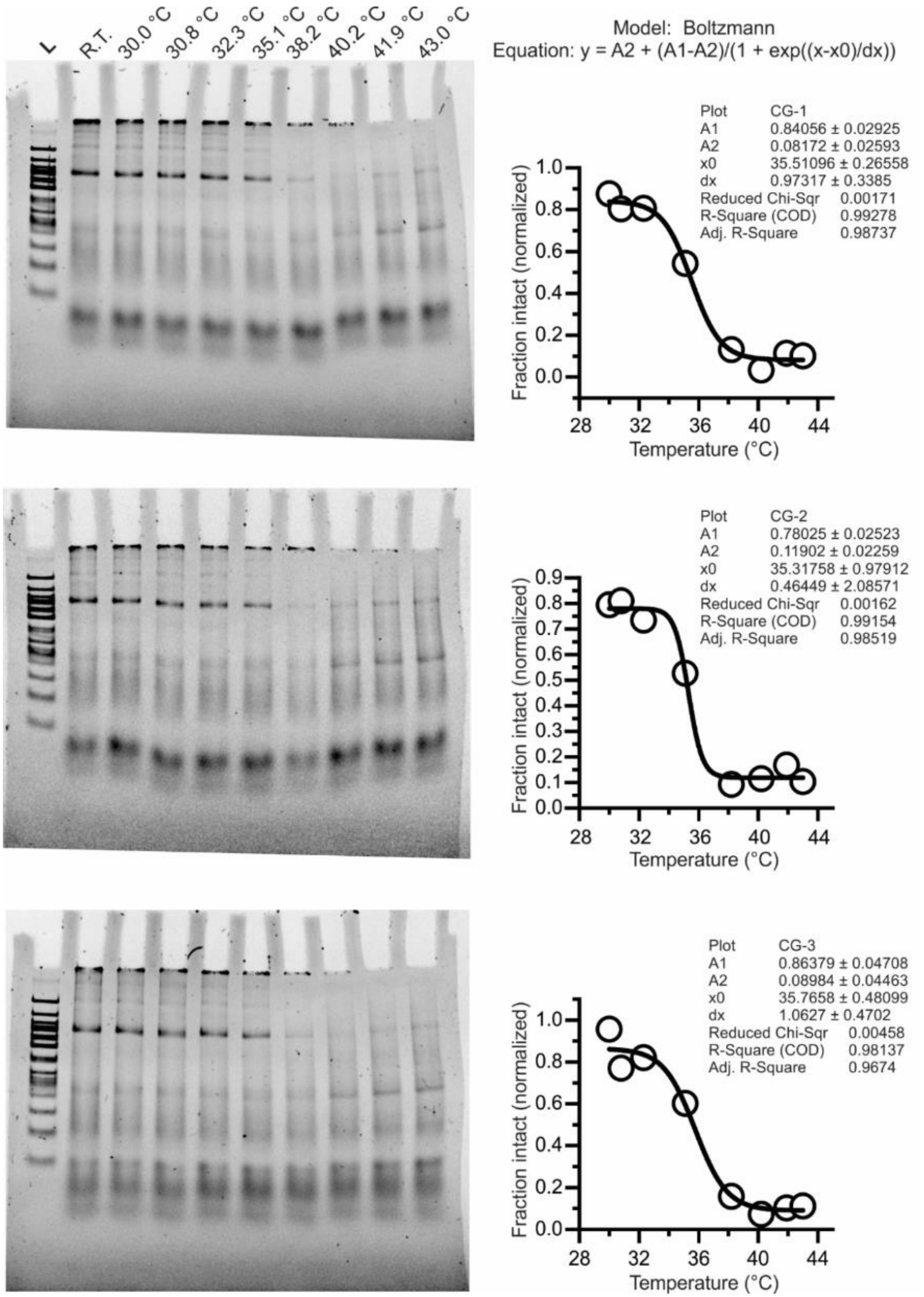

Figure S9: Triplicate melting experiments with tetrahedron with a C|G stack with 6 nucleotide sticky end. The formed nanostructures were incubated at various temperatures for 1 hour as indicated in the lane. The intact fraction of tetrahedron was fitted with Boltzmann model to obtain the melting temperature for each experiment.

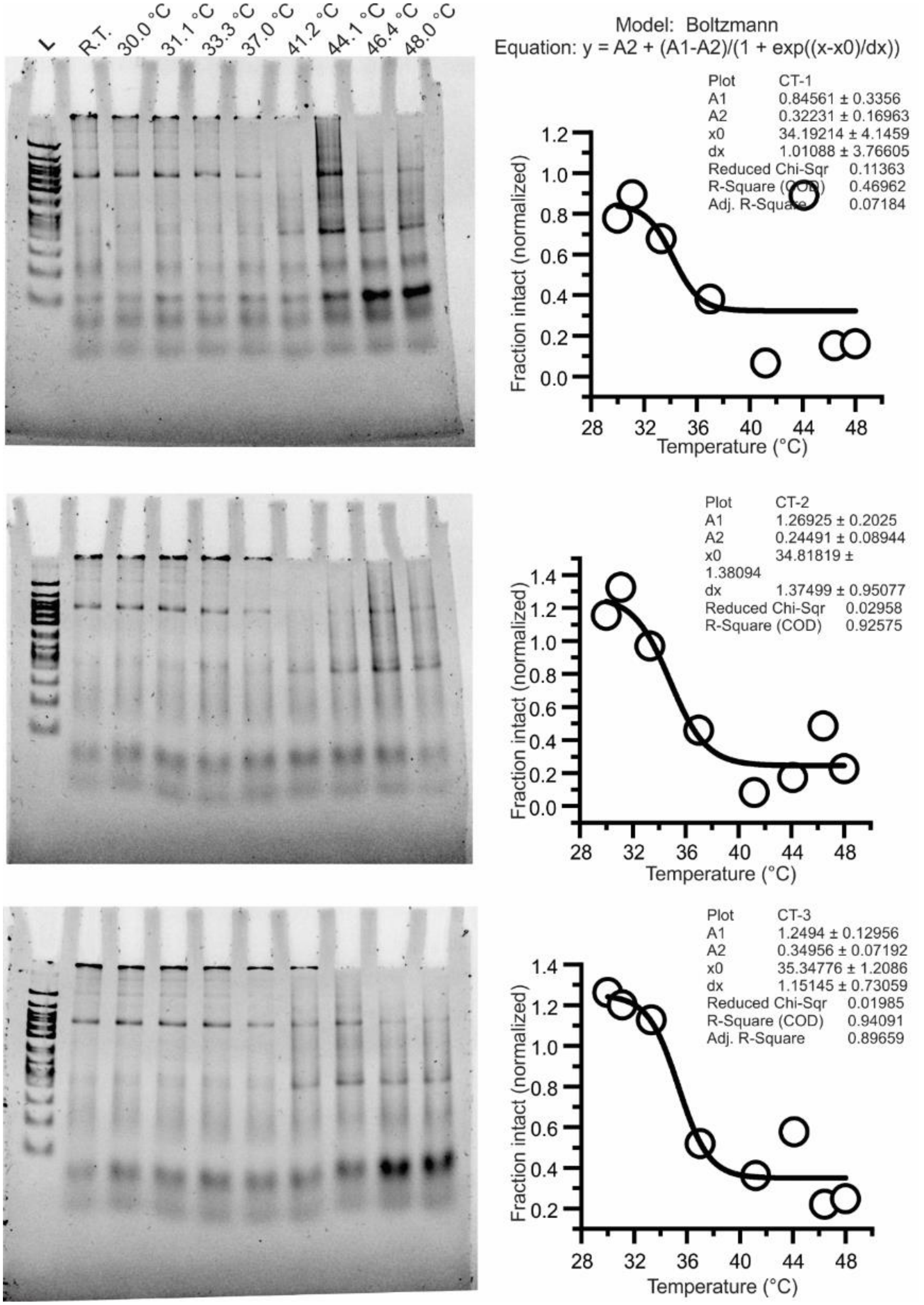

Figure S10: Triplicate melting experiments with tetrahedron with a C|T stack with 6 nucleotide sticky end. The formed nanostructures were incubated at various temperatures for 1 hour as indicated in the lane. The intact fraction of tetrahedron was fitted with Boltzmann model to obtain the melting temperature for each experiment.

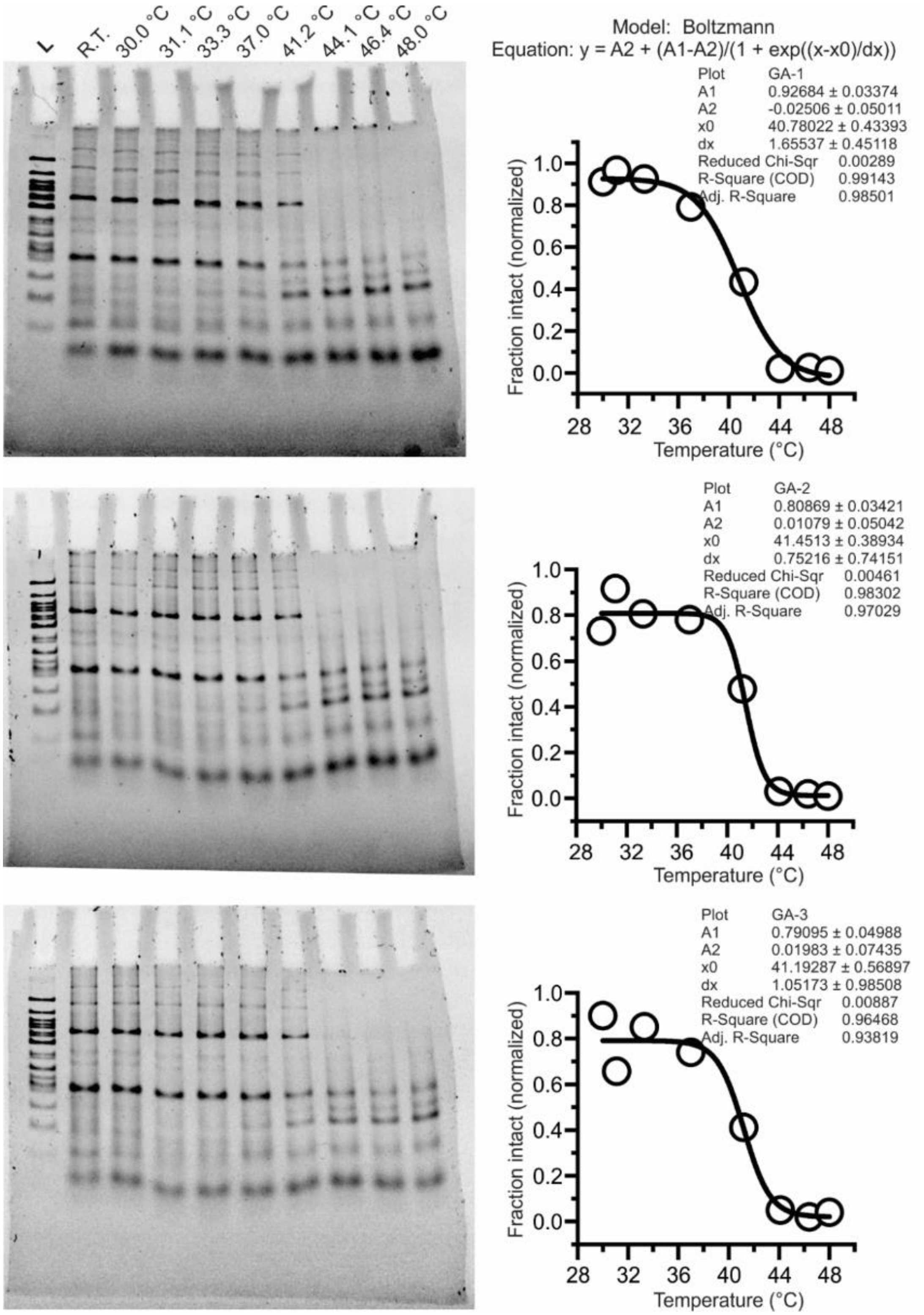

Figure S11: Triplicate melting experiments with tetrahedron with a G|A stack with 6 nucleotide sticky end. The formed nanostructures were incubated at various temperatures for 1 hour as indicated in the lane. The intact fraction of tetrahedron was fitted with Boltzmann model to obtain the melting temperature for each experiment.

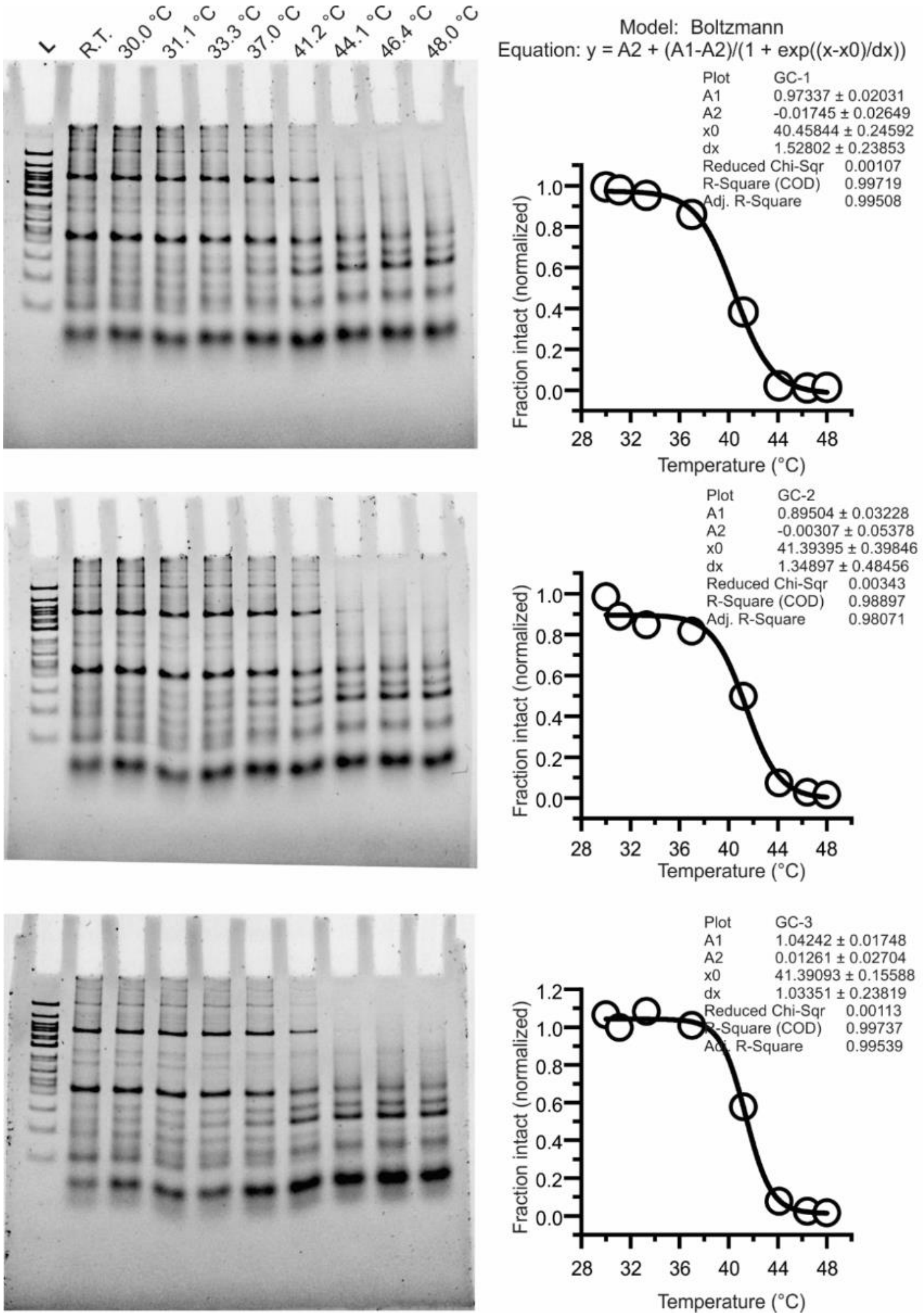

Figure S12: Triplicate melting experiments with tetrahedron with a G|C stack with 6 nucleotide sticky end. The formed nanostructures were incubated at various temperatures for 1 hour as indicated in the lane. The intact fraction of tetrahedron was fitted with Boltzmann model to obtain the melting temperature for each experiment.

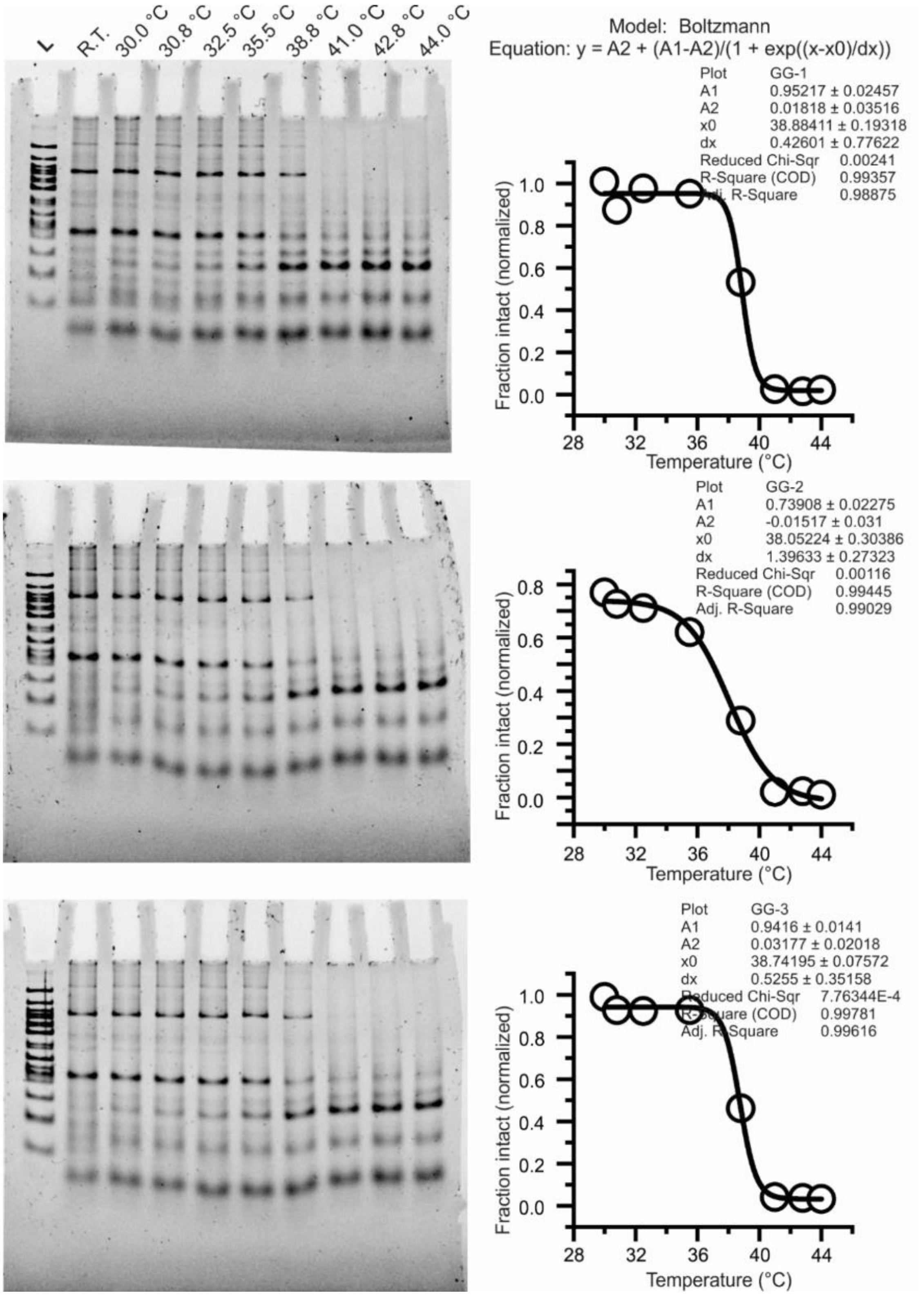

Figure S13: Triplicate melting experiments with tetrahedron with a G|G stack with 6 nucleotide sticky end. The formed nanostructures were incubated at various temperatures for 1 hour as indicated in the lane. The intact fraction of tetrahedron was fitted with Boltzmann model to obtain the melting temperature for each experiment.

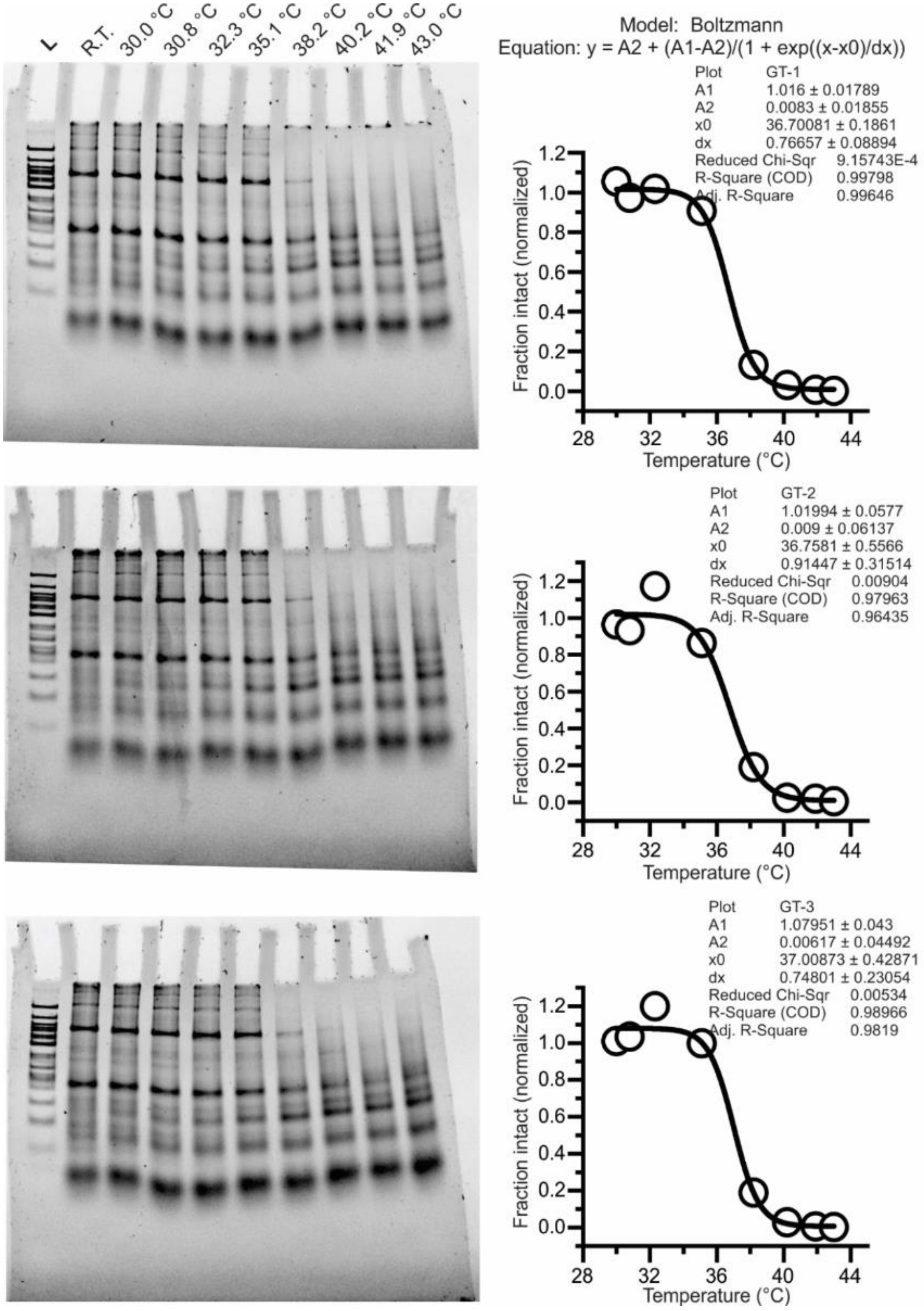

Figure S14: Triplicate melting experiments with tetrahedron with a G|T stack with 6 nucleotide sticky end. The formed nanostructures were incubated at various temperatures for 1 hour as indicated in the lane. The intact fraction of tetrahedron was fitted with Boltzmann model to obtain the melting temperature for each experiment.

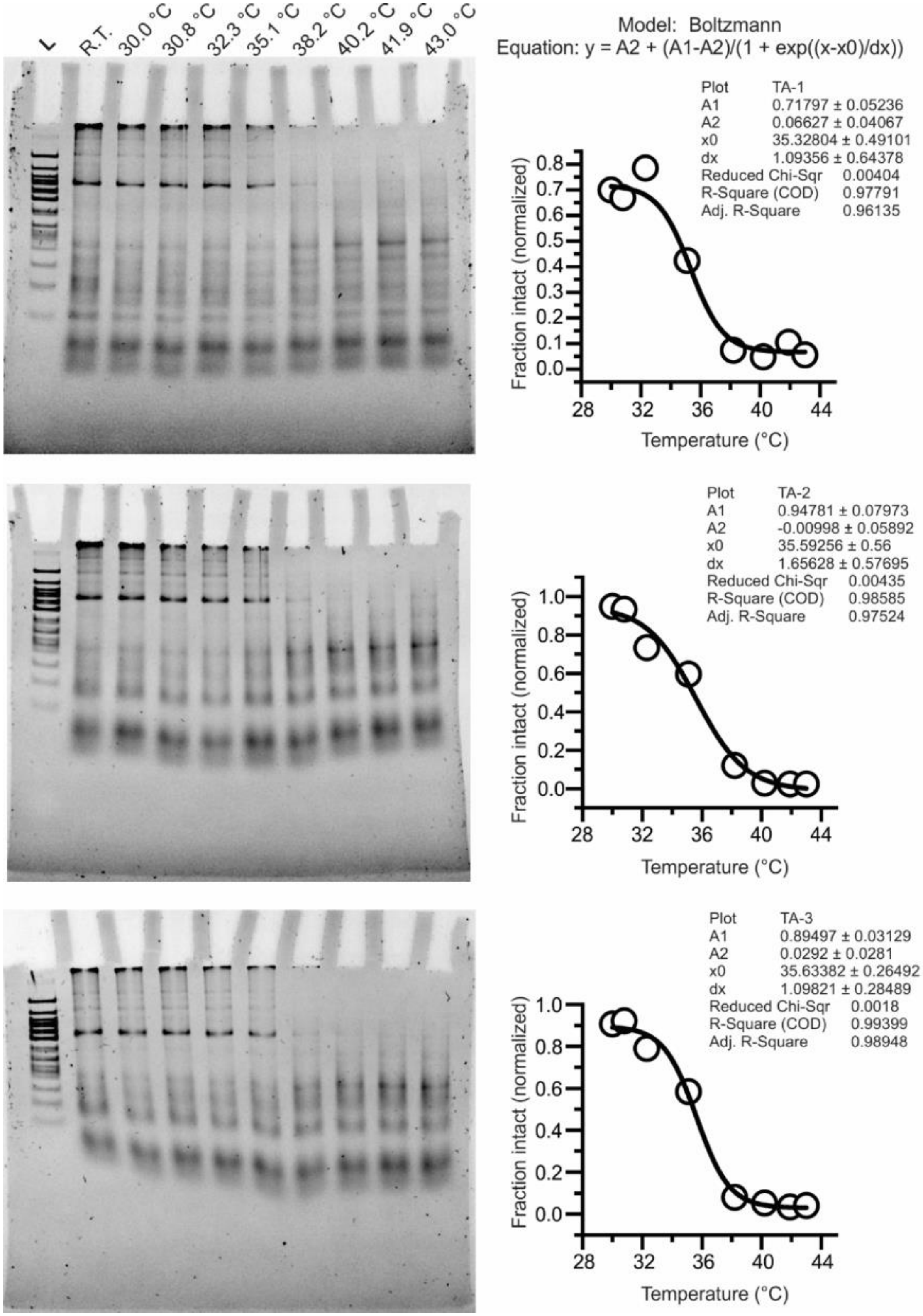

Figure S15: Triplicate melting experiments with tetrahedron with a T|A stack with 6 nucleotide sticky end. The formed nanostructures were incubated at various temperatures for 1 hour as indicated in the lane. The intact fraction of tetrahedron was fitted with Boltzmann model to obtain the melting temperature for each experiment.

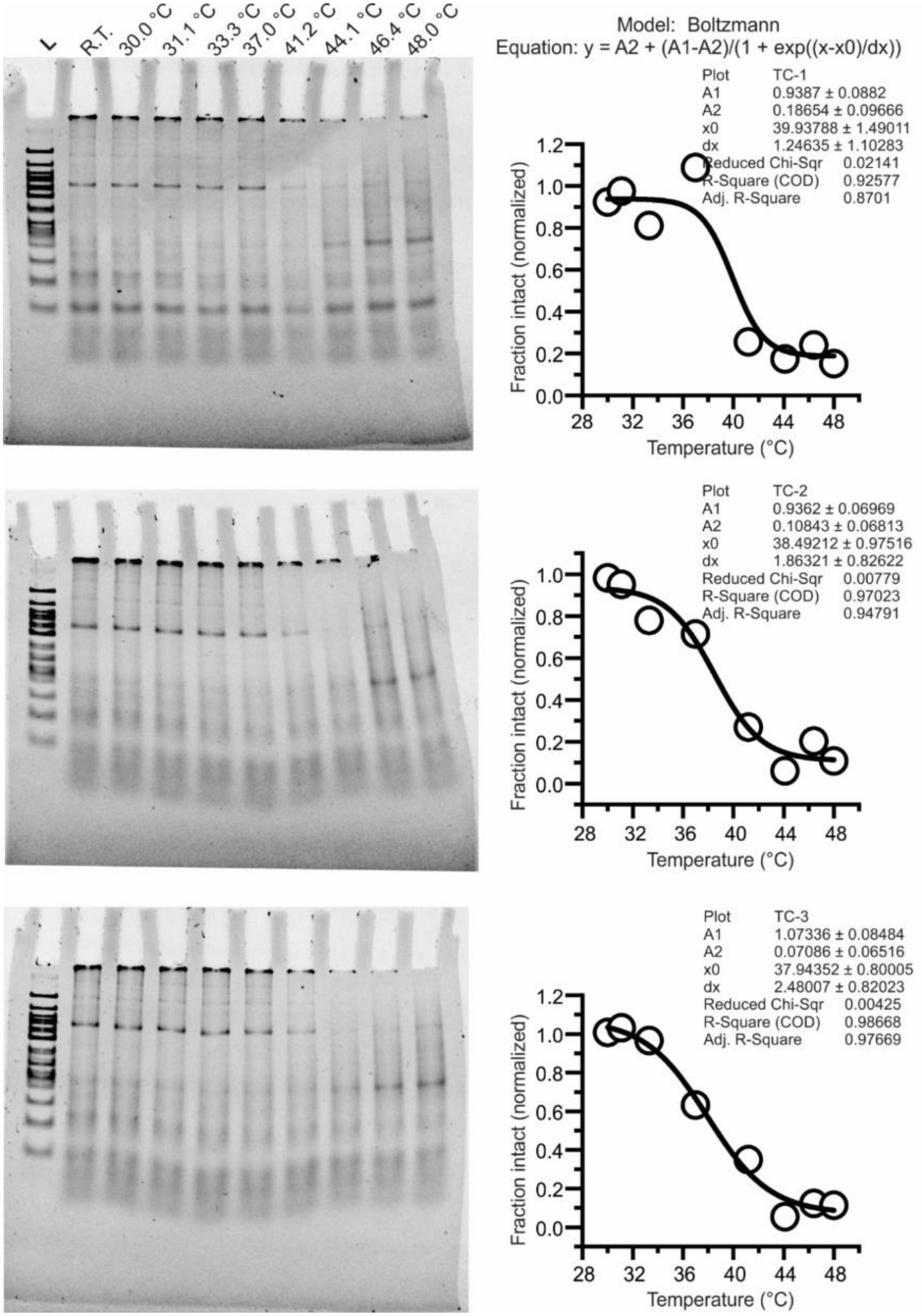

Figure S16: Triplicate melting experiments with tetrahedron with a T|C stack with 6 nucleotide sticky end. The formed nanostructures were incubated at various temperatures for 1 hour as indicated in the lane. The intact fraction of tetrahedron was fitted with Boltzmann model to obtain the melting temperature for each experiment.

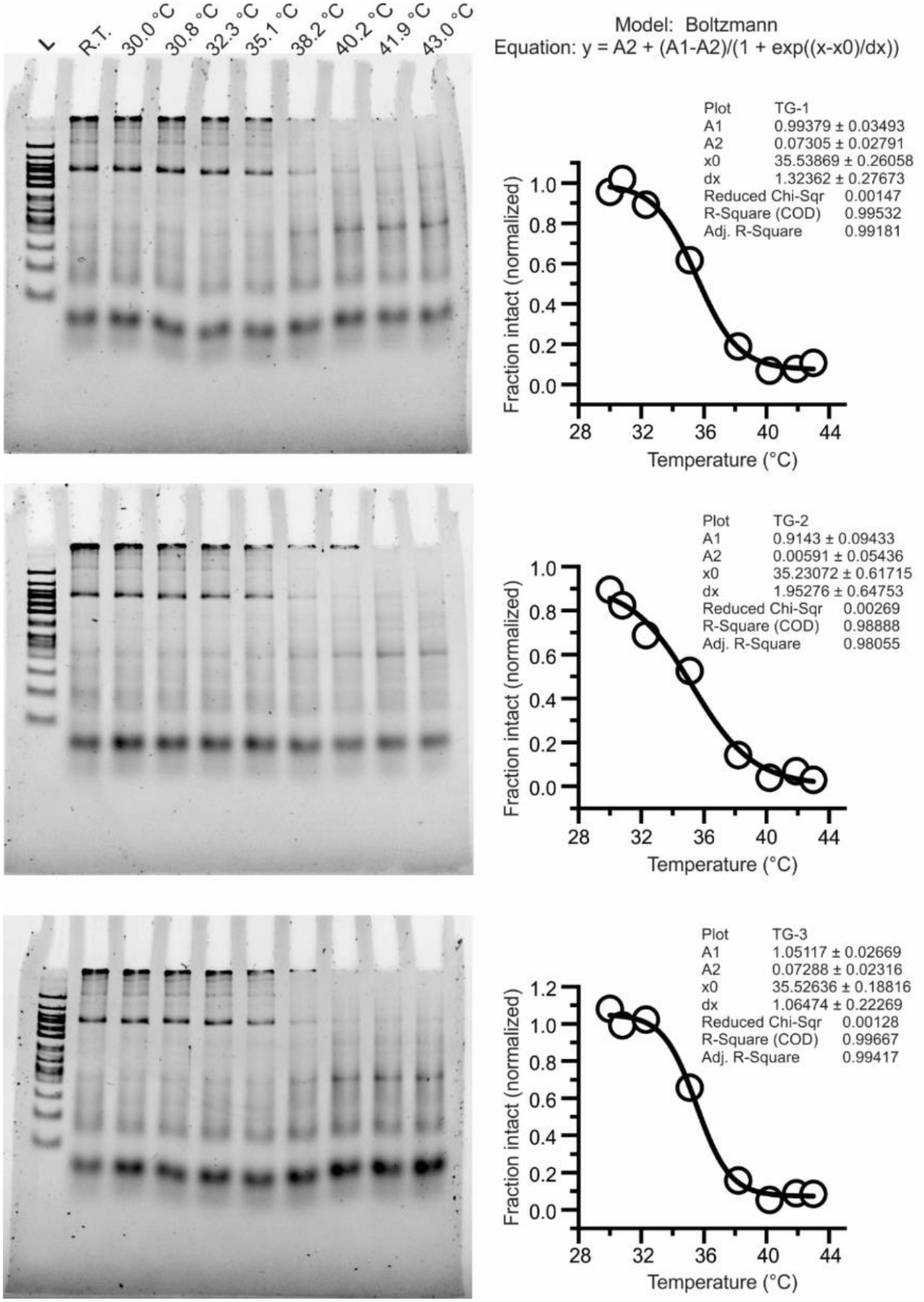

Figure S17: Triplicate melting experiments with tetrahedron with a T|G stack with 6 nucleotide sticky end. The formed nanostructures were incubated at various temperatures for 1 hour as indicated in the lane. The intact fraction of tetrahedron was fitted with Boltzmann model to obtain the melting temperature for each experiment.

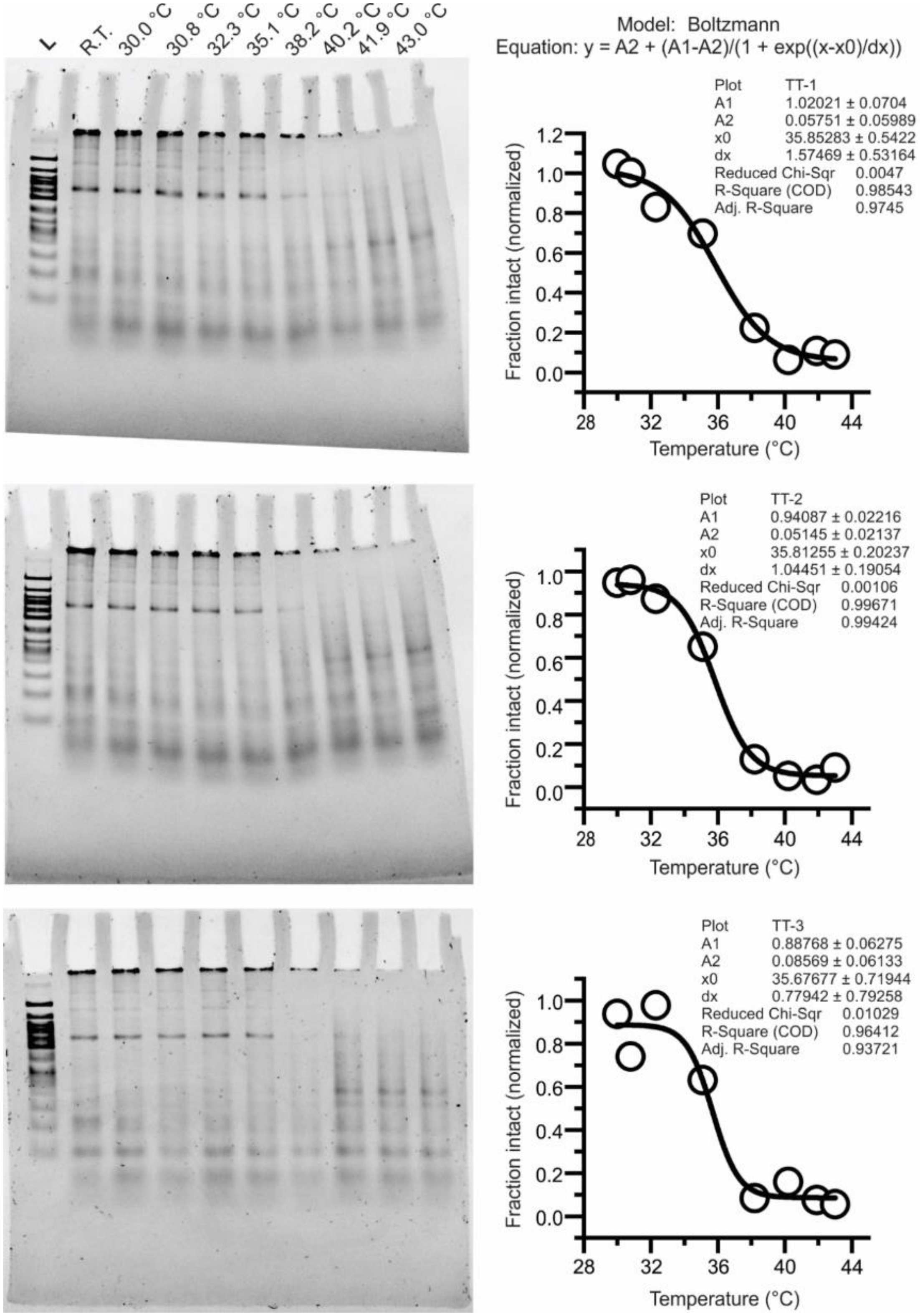

Figure S18: Triplicate melting experiments with tetrahedron with a T|T stack with 6 nucleotide sticky end. The formed nanostructures were incubated at various temperatures for 1 hour as indicated in the lane. The intact fraction of tetrahedron was fitted with Boltzmann model to obtain the melting temperature for each experiment.

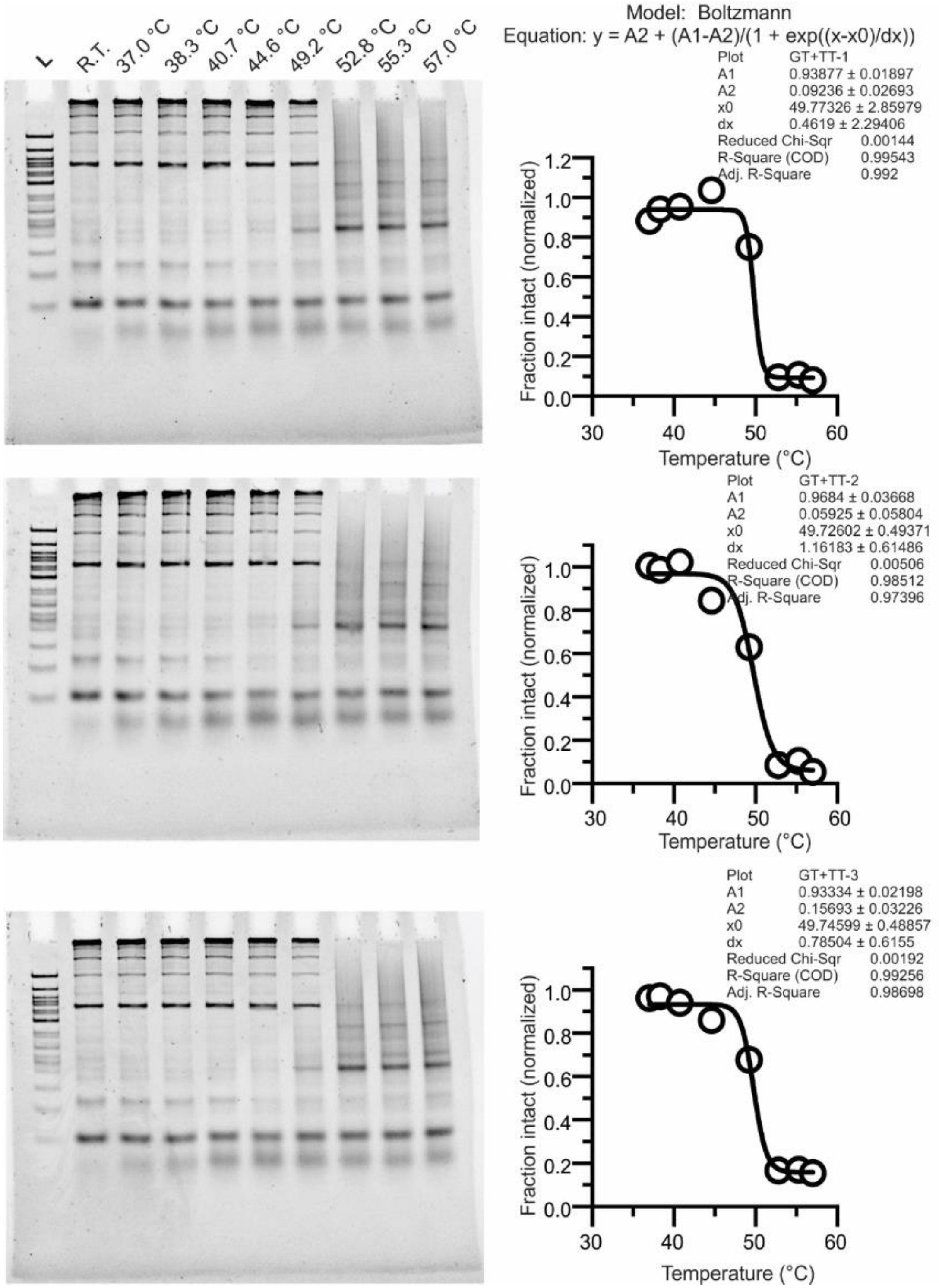

Figure S19: Triplicate melting experiments with tetrahedron with a G|T and T|T stack with 6 nucleotide sticky end. The formed nanostructures were incubated at various temperatures for 1 hour as indicated in the lane. The intact fraction of tetrahedron was fitted with Boltzmann model to obtain the melting temperature for each experiment.

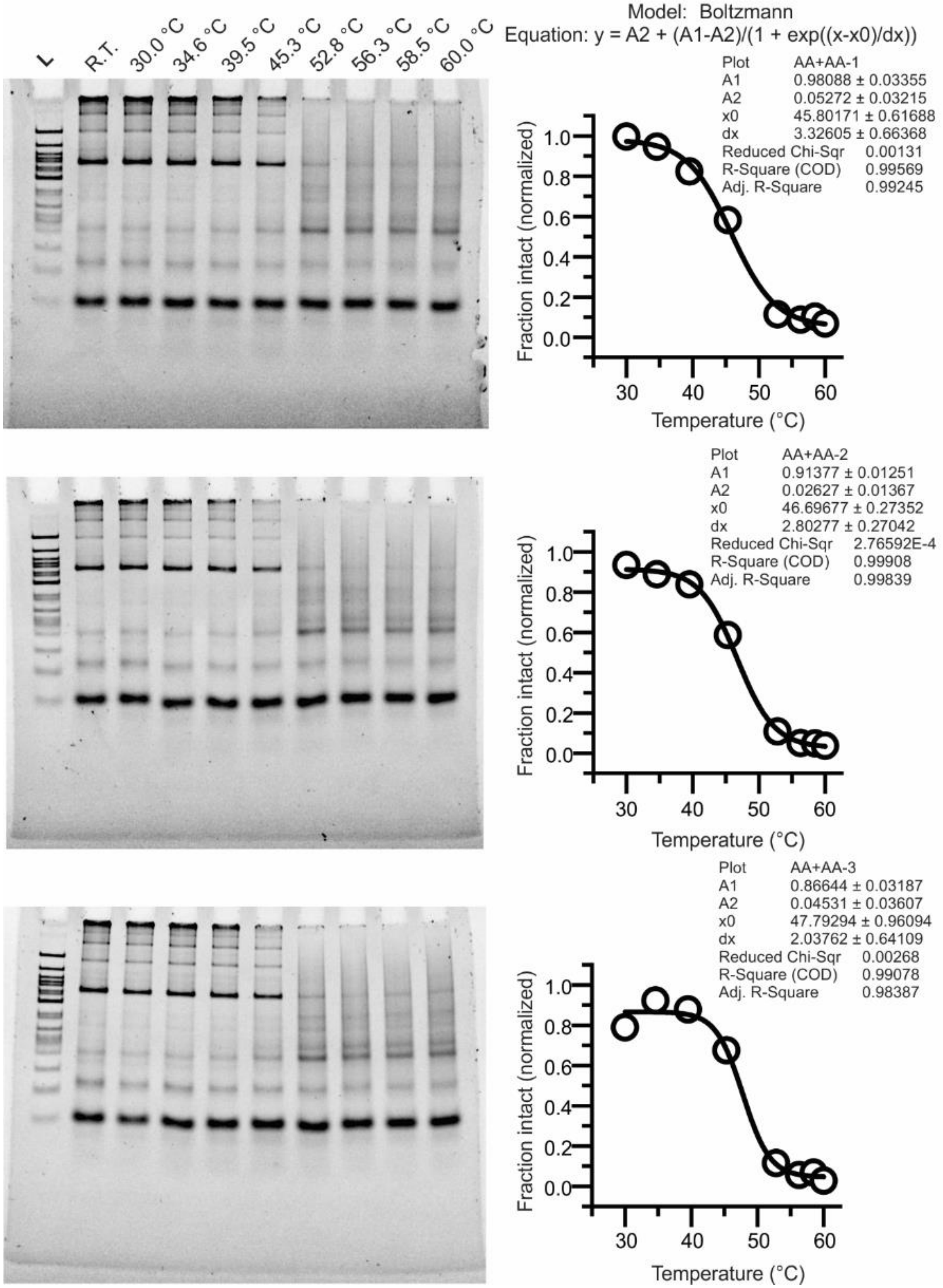

Figure S20: Triplicate melting experiments with tetrahedron with 2 A|A stack and a 4 nucleotide sticky end. The formed nanostructures were incubated at various temperatures for 1 hour as indicated in the lane. The intact fraction of tetrahedron was fitted with Boltzmann model to obtain the melting temperature for each experiment.

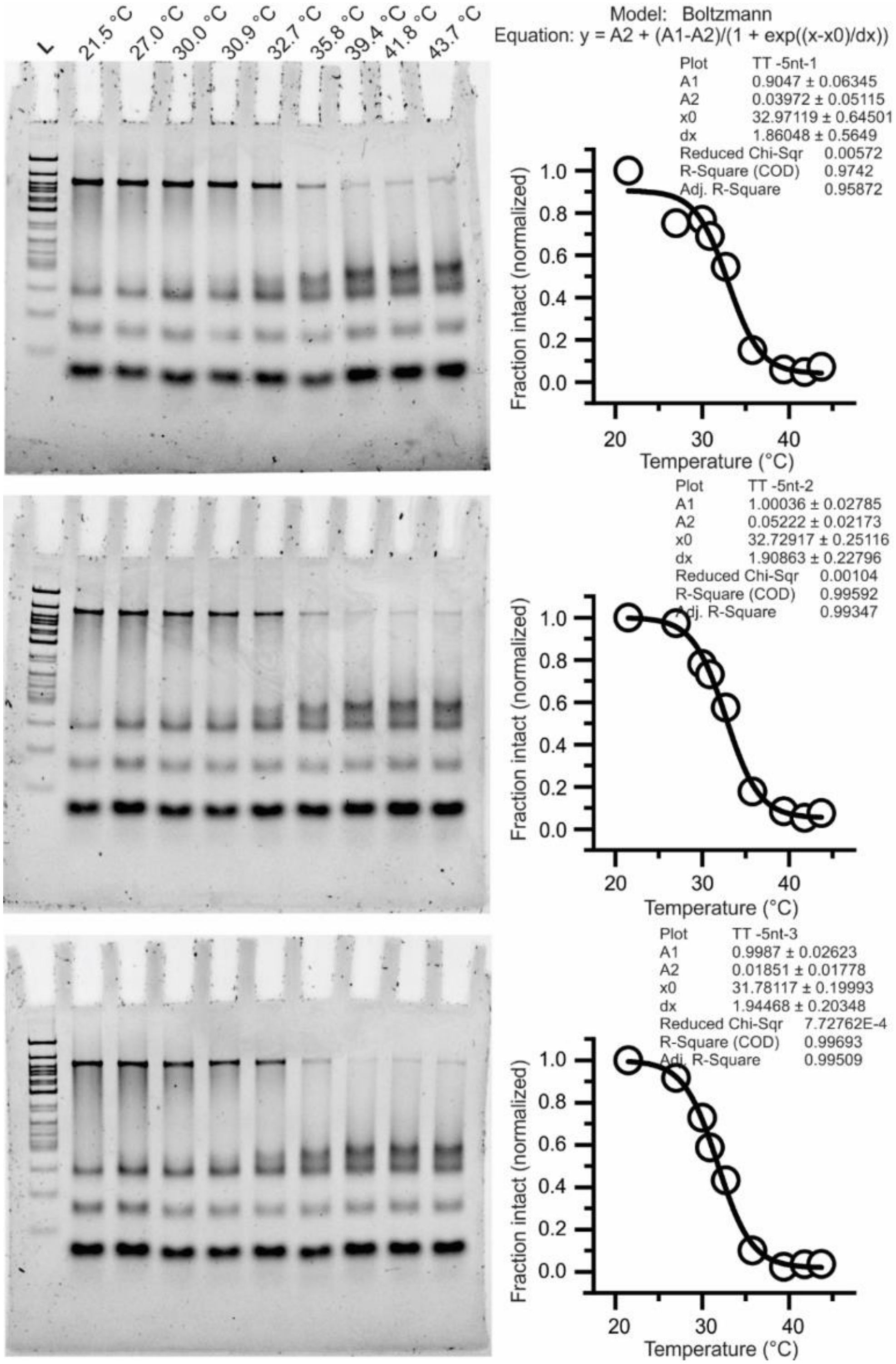

Figure S21: Triplicate melting experiments with tetrahedron with 1 T|T stack and a 5 nucleotide sticky end. The formed nanostructures were incubated at various temperatures for 1 hour as indicated in the lane. The intact fraction of tetrahedron was fitted with Boltzmann model to obtain the melting temperature for each experiment.

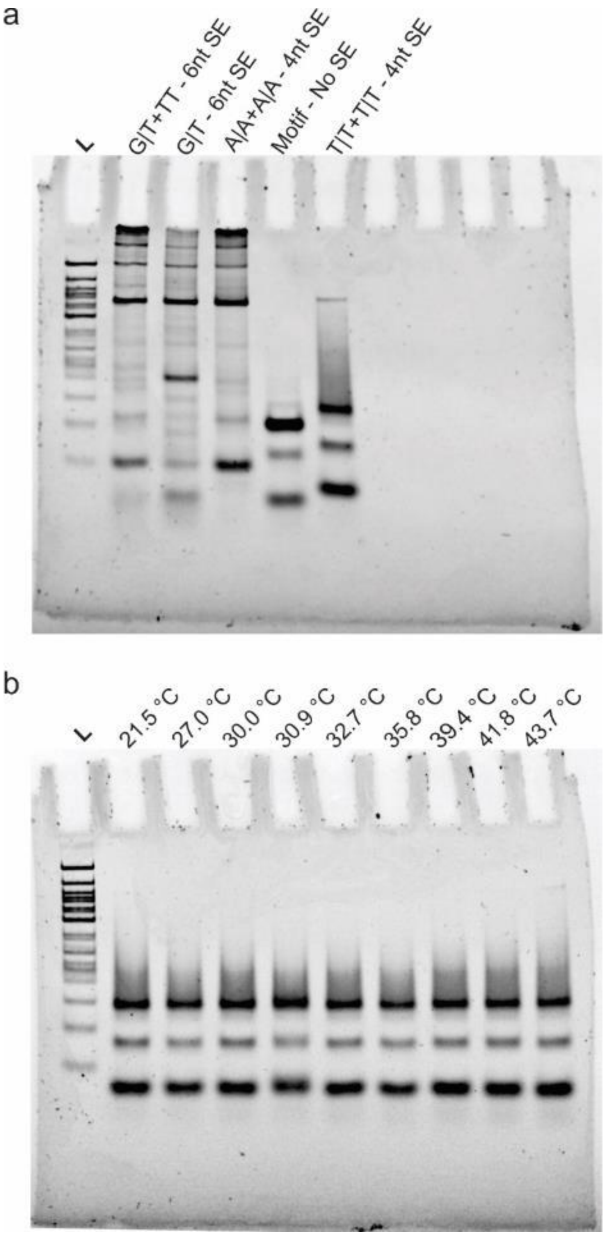

Figure S22: Tetrahedron with 6 and 4 nt sticky ends with different base-stacks. (a) PAGE confirmation formation of tetrahedra. Lane 1: 100 bp ladder, Lane 2: Tetrahedron with 6nt sticky end with G|T and T|T base stacks that are considered to be weak. Lane 3: Tetrahedron with 6nt sticky end with a single G|T stack. Lane 4: Tetrahedron with 4nt sticky end with 2 A|A stacks, which is the strongest stacks. Lane 5: DNA motif without sticky-end. Lane 6: Tetrahedron with 4nt sticky end with 2 T|T stacks, which is one of the weakest stacks and does not form reliably. (b) The tetrahedron with 4nt sticky ends and two T|T stacks was weak and not quantifiable during melting point determination.

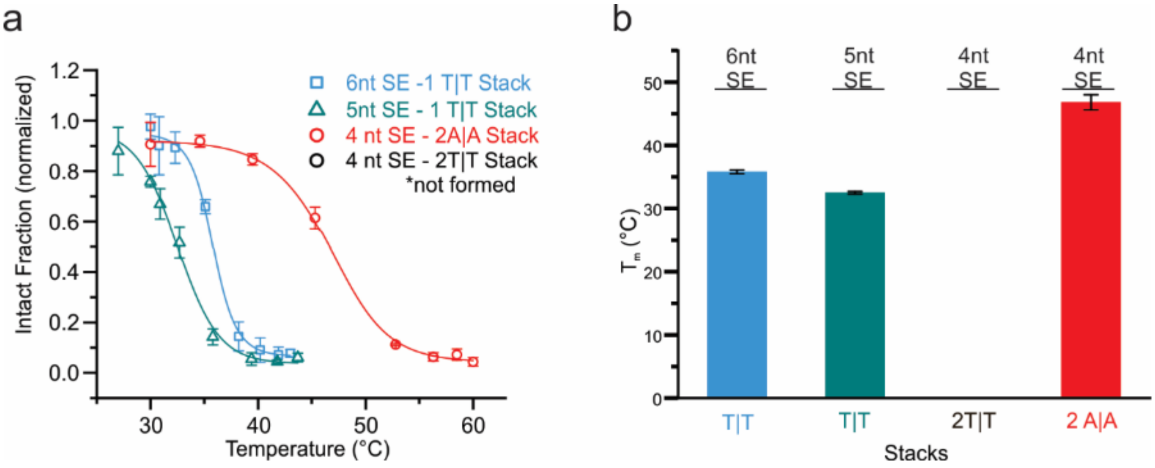

Figure S23: Thermal stability difference between tetrahedron with T|T stacks and A|A stacks. (a) The average melting profiles of the 3 tetrahedra that formed from triplicate measurements (Figures S18, S21, S22), fit with a Boltzmann curve to determine the melting temperatures. Tetrahedra with 4nt SE with 2 T|T stack failed to assemble (Figure S22), hence melting temperature wasn't determined. (b) A bar graphs representing the average meting temperatures of a 5 and 6 nt sticky end tetrahedron with a T|T stack and 4 nt sticky end tetrahedron with A|A stacks. The 4nt tetrahedron with 2 T|T stack failed to assemble. The error bars represent propagated error from the individual fits from Figure S18, S21 and S22. These experiments show that the reduced binding energy from shortened sticky-end can be compensated with stronger stacks.

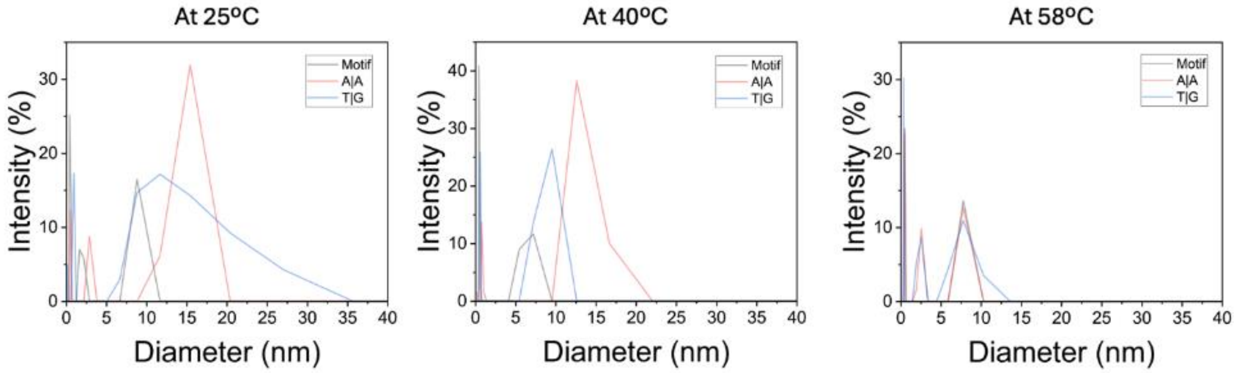

Figure S24: Dynamic light scattering of tetrahedra with T|G and A|A stacks. DLS measurements of blunt end tile (motif), weak stacking tetrahedron (T|G), and strong stacking tetrahedron (A|A) at different temperatures. At room temperature, the blunt end tile shows a peak below 10 nm, the A|A tetrahedron shows a single sharp peak near 15 nm, and the T|G shows a broad distribution in the 5-25 nm range. At 40˚C The A|A tetrahedron is minimally disturbed, while the T|G tetrahedron shifted to a single peak below 10 nm consistent with the motif. At 58˚C, all three have similar size distributions below 10 nm.
